# Job stress and depression among Malaysian anti-drug professionals: The moderating role of job-related coping strategies

**DOI:** 10.3389/fpsyt.2022.1020947

**Published:** 2022-11-07

**Authors:** Noradilah Md Nordin, Naqi Dahamat Azam, Mohd Roslan Rosnon, Mansor Abu Talib

**Affiliations:** ^1^Centre for Human Sciences, Universiti Malaysia Pahang, Kuantan, Malaysia; ^2^Department of Human Development and Family Studies, Faculty of Human Ecology, Universiti Putra Malaysia, Serdang, Malaysia; ^3^Department of Social and Development Sciences, Faculty of Human Ecology, Universiti Putra Malaysia, Serdang, Malaysia; ^4^Faculty of Social Science and Liberal Arts, UCSI University, Kuala Lumpur, Malaysia

**Keywords:** mental health problem, depression, control coping, avoidant coping, Malaysia

## Abstract

Depression can cause negative consequences to workers' health and social functioning, such as poor work productivity, mental disorders, and suicide. Existing studies have argued that job stress is closely related to depression in many professions. Yet, information on how coping strategies play a significant role in the relationships among Malaysian anti-drug professionals is still scarce. Thus, the aim of this study was to examine to what extent coping strategies moderate the relationship between job stress and depression among Malaysian anti-drug professionals. A total of 3,356 National Antidrug Agency (NADA) officers aged between 21 and 59 years completed online self-report measures of depression, job stress, and job-related control coping and avoidant coping behaviors. The results showed that job stress was strongly correlated with depression, and both coping strategies were found to significantly moderate the correlations. The correlations between stress and depression were stronger among participants who had higher levels of avoidant coping or those who had lower levels of control coping. To conclude, this study highlights the importance of considering job stress and coping behaviors to understand anti-drug professionals' mental health during this challenging COVID-19 pandemic.

## Introduction

Depression is a critical mental health issue that has attracted much attention among Malaysians in recent years. It is regarded as the leading psychological cause of suicidality and suicidal ideation in Malaysia (1–3). Moreover, depression can have negative consequences for individual physical, social, and emotional functioning. Physically, depressed people usually have problems with insomnia, constant tiredness, loss of appetite, and substance use (4–6). Also, they often avoid social interaction, which increases loneliness and social isolation (7–9). Regarding emotions, depressed people tend to experience low moods, such as persistent feeling of sadness (10–12).

Much effort has been devoted to understand depression and its underlying factors among different age groups in Malaysia. For instance, research has identified family background, lifestyle factors, bullying experiences, sleeping problems, and self-esteem as main risk factors of depression in young age groups, such as school and university students (13–15). Meanwhile, in adult working populations, some major contributors to depression are high job demands and poor working conditions (16, 17). While job stress has been long identified as closely linked to depression globally (18, 19), this association is less evident in Malaysia.

Job stress refers to the physical and emotional (negative) outcomes that result from a worker's inability to conform to the job demands and requirements (20, 21). Commonly, law enforcement personnel such as policemen and correctional officers are more likely to experience higher job stress than other occupational groups (22, 23). Furthermore, evidence shows that intensive job stress experienced by law enforcement personnel can lead to depression (24–26). Yet, thus far, no study in Malaysia has investigated the relationship between job stress and depression among law enforcement personnel.

Coping is also an important predictor of depression (27). It refers to physical and mental efforts used to deal with stressful situations (28). Latack (29) suggested two strategies that can be applied to cope with job stress, namely control and avoidant coping. Control coping refers to a problem-focused strategy meant to address the root causes of a stressful situation in a healthy way. This strategy involves altering a stressor or one's reaction to the stressor. Meanwhile, avoidant coping is a passive coping strategy in which individuals rarely make efforts to change stressful situation (30). Among the common strategies used for avoidant coping are ignoring the stressor and withdrawing from the stressful situation. Although avoidant coping might be a good ‘short-term' solution, unsolved problems can potentially elicit more conflicts and distress in interpersonal relationships and possibly other functioning, thus maintaining depression in the long run (31). Furthermore, previous studies have suggested that individuals' preferences for coping strategies can have an impact on psychological disorders, such as depression. Individuals who employs control coping strategies are able to actively and constructively address their stressors, which can lessen the likelihood of depression (32, 33). However, individuals who prefer to avoid solving problems may tend to be depressed, due to their ineffectiveness in dealing with the stressors (31, 34).

Thus far, enormous efforts have been devoted to understand the relationship between stress and depression and how preferences for coping strategies differ in that relationship. Yet, less attention has been paid on this topic in Malaysian law enforcement agencies, particularly the National Antidrug Agency (NADA). As the name implies, NADA is a Malaysian government agency that functions primarily to curb drug and substance abuse through prevention, treatment, and rehabilitation (35). Yet, despite NADA achieving the highest recovery rate of drug addicts in 2018, the nature of the organization's operation is undeniably involving high risk activities. For instance, NADA officers are exposed to violence and infectious diseases carried by illicit drug users who are aggressive (36, 37). Existing studies have shown that professionals who work in the community are at risk of developing psychological distress such as depression and post-traumatic stress syndrome when experiencing intense stressors at work (38, 39). Thus, coping strategies are essential to protect these professionals' mental health well-being and improve their job performance (40).

Therefore, the aim of this study was to examine the relationships between job stress, coping strategies, and depression among NADA officers. Regarding this, it was expected that job stress and avoidant coping would be related to more depression, and control coping would be related to less depression. Also, this study investigated any moderating effects of control and avoidant coping strategies on the relationship between job stress and depression among the participants. Due to this, it was expected that job stress would be related to higher levels of depression, particularly in participants who used less control coping and more avoidant coping.

## Methodology

### Study design, setting, and sample population

This study used a correlational cross-sectional research design in all zones of NADA (i.e., East and West Malaysia states). Due to the movement control order imposed in Malaysia during the COVID-19 outbreak, data were collected through an online Google Form survey. Prior to the data collection, ethical approval was granted by the Ethics Committee of Universiti Putra Malaysia. Also, permission was given by the management team of NADA to conduct this study, and informed consents were obtained from all participants. The NADA's Research Division was tasked to send the link to the online survey to the NADA staff's work email addresses. By using convenience sampling, a total of 3,356 NADA officers aged between 21 and 59 years (68.2% men; *m*_age_ = 37.92 years, *SD*_age_ = 7.01 years) participated in this study. The participants represented 57.4% of the total number of NADA employees (*N* = 5,838).

### Research tools

#### Socio-demographic questionnaire

The socio-demographic questionnaire asked about participants' sex, race, age, marital status, socioeconomic status (i.e., educational levels and monthly income), and years of service in the anti-drug profession.

#### Patient health questionnaire (PHQ)

The Malay version of the *Patient Health Questionnaire* [PHQ-9; (41)] is a 9-item, self-reported questionnaire that assesses the severity of depressive symptoms. Some examples of the items are “*Little interest or pleasure in doing things”* and “*Thoughts that you would be better off dead or of hurting yourself in some way.”* The participants were asked to rate their frequencies on each item on a Likert-type scale (0 = *not at all*, 1 = *several days*, 2 = *more than half the days*, 3 = *nearly every day*). The high score indicates high levels of depression.

#### Job-related tension index (JRTI)

The Malay version of *Job-Related Tension Index* (42, 43) is a 15-item self-reported questionnaire that measures participants' job stress. A few examples of the items “*Not knowing just what the people you work with expect of you*” and “*Feeling that your job tends to interfere with your family life*.” All items were rated on a 5-point Likert scale (from 1 = *never* to 5 = *rather often*). A high score indicates an individual's high job stress level.

### Coping with job stress

*Coping with Job Stress* (29) is a 28-item self-reported questionnaire that assesses two different coping behaviors, namely, control coping and avoidant coping. An example of a control coping item is “*Try to see this situation as an opportunity to learn and develop new skills*,” and an example of an avoidant-coping item is “*Separate myself as much as possible from the people who created this situation*.” The participants were asked to rate their response on each item with a 5-point scale (from 1 = *hardly ever do this* to 5 = *almost always do this*).

Recently, no Malay-translated version was available for Coping With Job Stress. Therefore, the questionnaire was translated from English into Malay (i.e., the national language of Malaysia), and it was back-translated by an independent bilingual translator. We compared the back-translated version with the original to check for language consistency, and any inconsistencies were resolved through discussion. We tested all questionnaires in a pilot study (*n* = 74) and minor amendments were made prior to conducting the actual study.

Table 1 presents the psychometric properties of all measures. Overall, the internal consistency was adequate (0.84 < α < 0.92).

**Table 1:** Psychometric properties of the questionnaires for depression, job stress, control coping and avoidant coping (*n* = 3356).

	***n* (%)**	***n* items**	**Range**	**Cronbach's α**	**Total**	**M (SD)**		***t***
						**Men** **(*n* = 2320)**	**Women** **(*n* = 1036)**	
Depression		9	0–3	0.87	8.43 (5.55)	8.52 (5.73)	8.24 (5.14)	1.38
Severed	149 (4.4)							
Moderately severed	336 (10.0)							
Moderate	658 (19.6)							
Mild	1339 (40.0)							
Minimal	869 (26.0)							
Job stress		15	1–3	0.94	2.53 (0.88)	2.54 (0.91)	2.50 (0.83)	1.16
High	1628 (48.6)							
Low	1723 (51.4)							
Coping strategies								
Control		17	1–5	0.92	3.96 (0.62)	3.97 (0.64)	4.00 (0.58)	−1.36
High	1723 (51.2)							
Low	1633 (48.7)							
Avoidant		11	1–5	0.84	2.88 (0.72)	2.89 (0.74)	2.86 (0.66)	1.04
High	1764 (52.6)							
Low	1592 (47.4)							

*p < 0.001.

### Statistical analyses

First, we performed descriptive analyses on each study variable. Second, we tested the relationships between job stress, control coping, avoidant coping, and depression using Pearson's correlation analysis. Third, we conducted two separate hierarchical linear regression analyses to examine the moderating role of coping strategies in any relationship between job stress and depression. In each analysis, the controlled variable (age) was entered in the first step (Model 1), the independent variables (job stress) and moderating variables (control coping, avoidant coping) in the second step (Model 2), and the interaction variables (job stress × control coping, job stress × avoidant coping) in the third step (Model 3). All analyses were conducted using IBM SPSS version 26 (44).

### Missing data analysis

Prior to data analysis, a missing value analysis was conducted to determine the proportion and pattern of missing data in our study. The results of Little's MCAR test (χ^2^ = 4.339, *DF* = 5, *p* = 0.503) indicated missing completely at random. Considering the small amount of missing data (0.07% incomplete cases and 0.01% unfilled values), we performed a complete case analysis (*list-wise deletion*) for all further analyses.

## Results

### Socio-demographic characteristics of the study participants

A total of 3,356 anti-drug professionals were selected as participants in the study. The majority of the participants were men (69.1%), Malays (88.4%), aged between 31 and 40 years (61.0%), and married (87.2%). Regarding socioeconomic status, the majority had educational levels of high schools or diplomas (79.9%) and an income range between RM2001 and RM3000 (50.1%). Also, 63.6% of participants had worked as an anti-drug officer for 11–20 years.

### Descriptive characteristics and levels of job stress, coping behaviors, and depression

Table 1 presents the descriptive characteristics of each study variable. Based on the findings, more than half of the participants reported low levels of stress but higher levels of control coping and avoidant coping. Also, the majority of the participants experienced mild and minimal levels of depression. Furthermore, our preliminary analysis showed gender similarities in job stress and depression levels, as well as the frequency with which both coping behaviors were used.

### Relationships between job stress, coping strategies, and depression

Table 2 shows the results of the Pearson's bivariate correlations of job stress, control coping, avoidant coping, and depression. The results showed that depression was positively related to job stress and avoidant coping, but negatively related to control coping. Furthermore, job stress was related to less control coping and more avoidant coping. Also, control coping was positively related to avoidant coping.

**Table 2:** Pearson's correlation coefficients of work stress and job coping strategies (control, avoidant) on depression (*n* = 3356).

	**Depression**	**Stress**	**Control coping**	**Avoidant coping**
**Depression**	-			
Stress	0.65^***^	-		
Control coping	−0.10^***^	−0.10^***^	-	
Avoidant coping	0.39^***^	0.39^***^	0.09^***^	-

*** *p* > 0.001.

### Control coping as the moderator

Table 3 depicts the results of the hierarchical regression analysis with depression as the dependent variable, job stress as an independent variable, and avoidant coping as a moderating variable. It was shown that younger participants reported more depression (Model 1). Moreover, higher levels of control coping were related to less depression, yet job stress and avoidant coping were related to more depression (Model 2). Furthermore, control coping interacted with the levels of job stress (Model 3).

**Table 3:** Regression analysis showing age, stress and control coping as predictors of depression (*n* = 3,356).

**Predictor**	**Depression**				
	**B**	**β**	***SE B***	***P***	***R^2^*/Δ*R^2^***
Model 1					0.01/0.01^*^
Age	−0.07	−0.10	0.01	0.000	
Model 2					0.43/ 0.42^*^
Stress	4.05	0.64	0.08	0.000	
Control	−0.55	−0.06	0.12	0.000	
Model 3					0.43 /0.00^*^
Stress × Control	−0.45	−0.32	0.12	0.000	

* *p* < 0.001.

β = standardized regression coefficients; SE, Standard Error; P, significant value; Δ R^2^ = change in R^2^ value.

**Figure 1:**
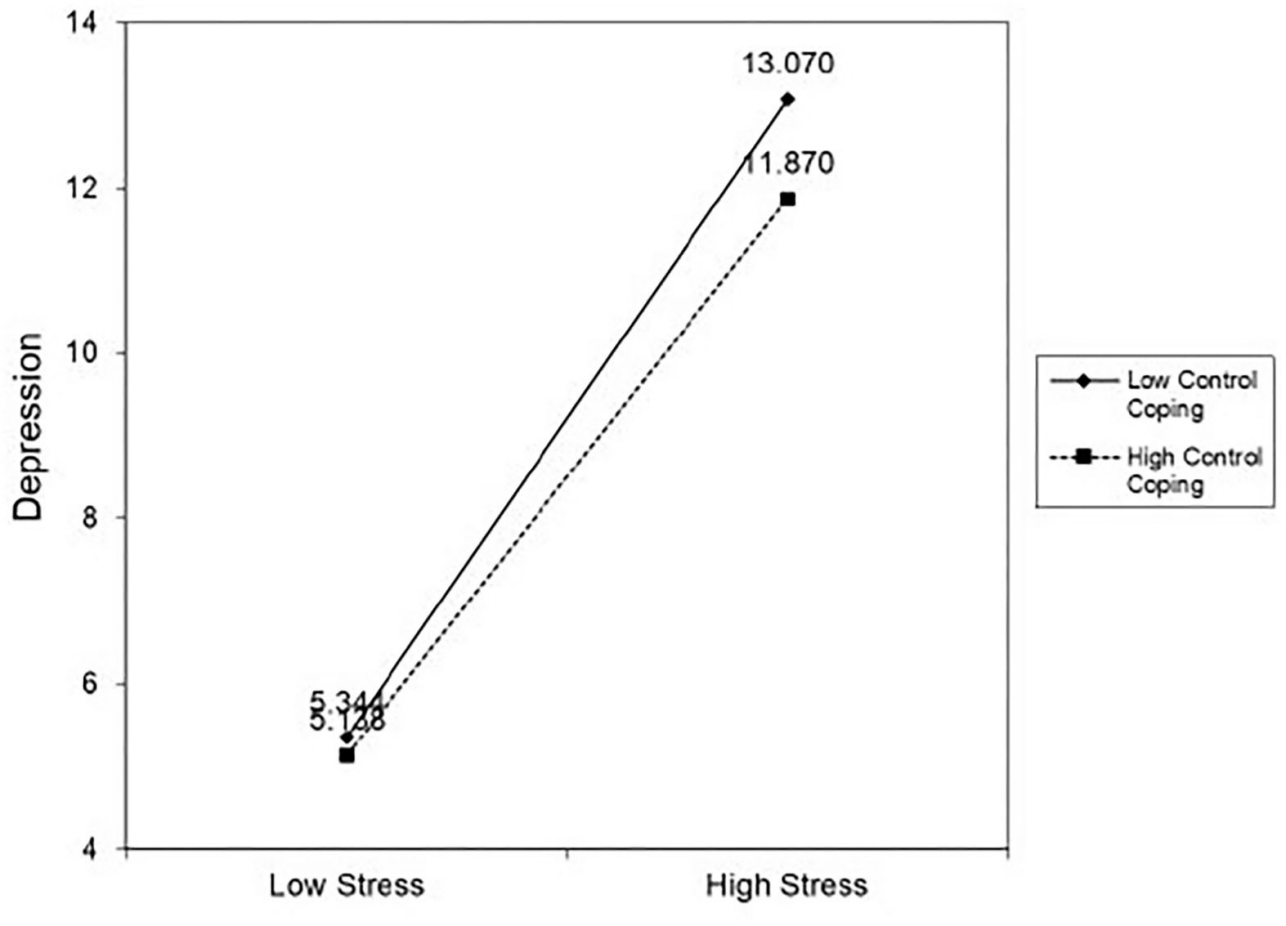
The moderating effect of control coping on the relationships between job stress and depression.

As shown in Figure 1, higher levels of job stress were related to depression in participants with any degree of control over coping. Yet, the effects were more pronounced in participants with lower levels of control coping. Supplementary Table 1 presents the results of a hierarchical regression analysis in greater detail.

### Avoidant coping as the moderator

Table 4 depicts the results of the hierarchical regression analysis with depression as the dependent variable, job stress as an independent variable, and avoidant coping as a moderating variable. It was shown that younger participants reported more depression (Model 1), and higher levels of stress predicted more depression (Model 2). Moreover, avoidant coping interacted with the levels of job stress (Model 3).

**Table 4:** Regression analysis showing age, stress, and avoidant coping as predictors of depression (*n* = 3356).

**Predictor**	**Depression**				
	**B**	**β**	***SE B***	***P***	***R^2^*/Δ*R^2^***
Model 1					0.01/0.01^*^
Age	−0.07	−0.10	0.01	0.000	
Model 2					0.43/0.42^*^
Stress	3.75	0.60	0.09	0.000	
Avoidant	0.83	0.11	0.12	0.000	
Model 3					0.44/0.00^*^
Stress x Avoidant	0.47	0.34	0.09	0.000	

* *p* < 0.001.

β = standardized regression coefficients; SE, Standard Error; P = significant value; Δ R^2^ = change in R^2^ value.

**Figure 2:**
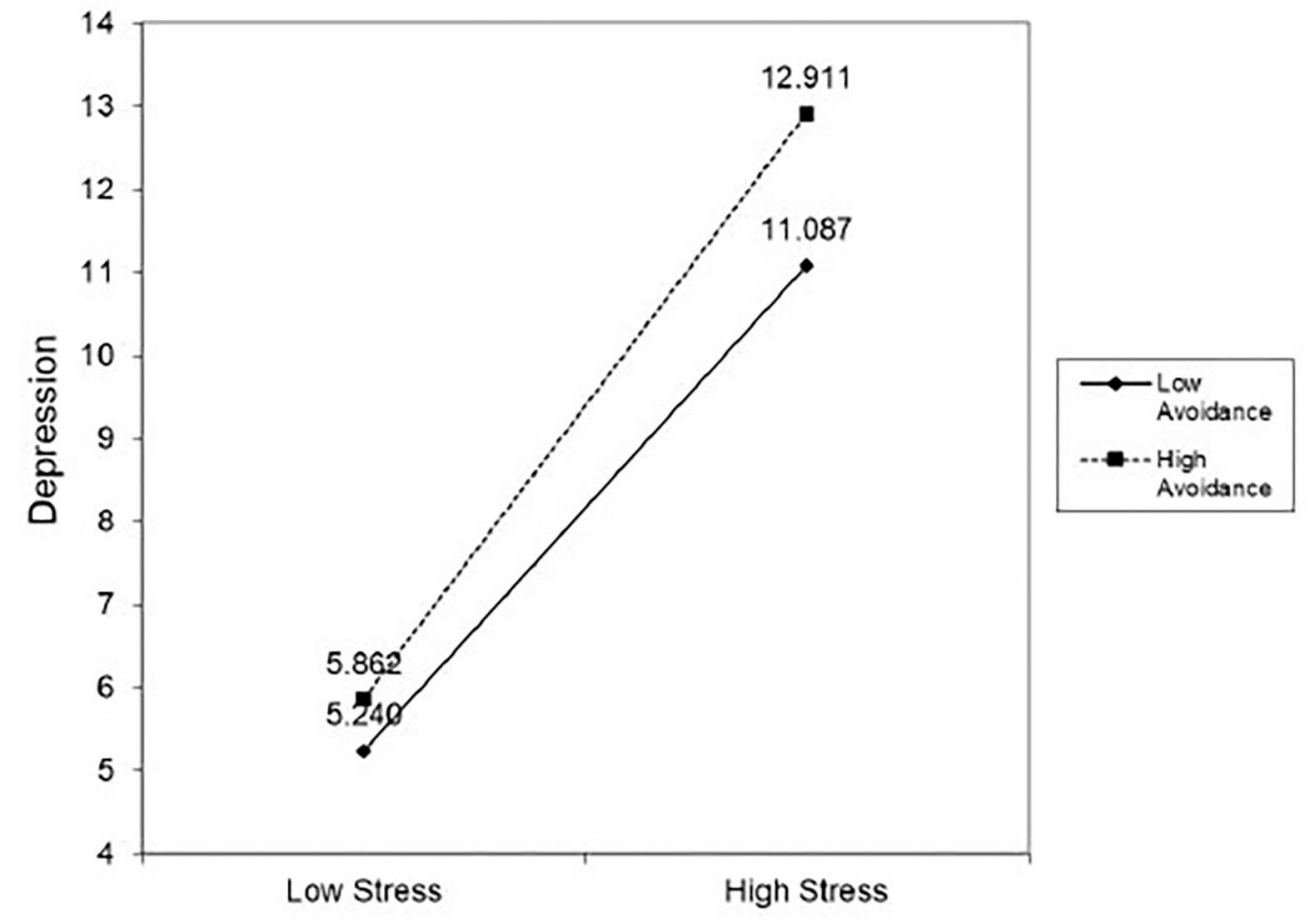
The moderating effect of avoidant coping on the relationships between job stress and depression.

As shown in Figure 2, higher levels of job stress were related to depression in participants with any degree of avoidant coping. Yet, the effects were more pronounced among participants with higher levels of avoidant coping. Supplementary Table 2 presents the results of a hierarchical regression analysis in greater detail.

## Discussion

The contribution of job stress and coping strategies to mental health issues, such as depression, has long since been studied. Understanding these relationships is indeed important and necessary to develop preventive strategies against depression in working populations. Yet, fewer studies have been conducted on the effect of different degrees of coping strategies on the relationships between job stress and depression, with far fewer studies available in East Asia. This study was focused on this.

In general, the findings from this study share many similarities with prior studies. First, as expected, job stress was related to more depression in anti-drug professionals. This finding supports the idea that job stress is a prominent factor of depression, which is now being witnessed in anti-drug professionals in Malaysia (24–31, 33–35, 38, 39). Indeed, considering the job nature of anti-drug professionals, which includes multiple responsibilities (i.e., enforcement, treatment, medication, and rehabilitation service) and contains occupational health risks (e.g., aggressive clients, infectious diseases among clients), it is understandable that the job itself poses a risk to their mental health well-being.

Also, in line with prior studies, control coping was related to less depression and stress, while avoidant coping was related to more depression and stress (31–34). These findings have strengthened the idea that control coping plays a better role as a buffer to psychological disorders than avoidant coping in anti-drug professionals. Indeed, by being active and positive in addressing problems at work, an individual can maintain good relationships and avoid conflicts with co-workers, thus reducing the chances of developing mental health problems (45). Meanwhile, ignoring and distancing self from stressful situations, which characterized avoidance strategies, did not help in solving problems, but also created more opportunities for stress and depression (46).

Furthermore, the expectations regarding the moderating effects of coping strategies were also met. As expected, the results showed that stress was related to more depression in anti-drug professionals who had lower levels of control coping and higher levels of avoidant coping. Certainly, individuals with poor control coping are more likely to experience anxiety, impulsiveness, and insecurity, but they are less likely to experience well-being (47, 48). Together, these negative properties of the self and avoidant coping strategies seem to not protect individuals from depression when intense stress is experienced.

Yet, unexpectedly and intriguingly, it was also found that anti-drug professionals who had higher levels of control coping and lower levels of avoidant coping also experienced depression when their stress levels were high. Commonly, both high-control coping and low-avoidant coping can be regarded as the properties of an adaptive and problem-focused approach to coping, which lead to better psychological outcomes (49, 50). Thus, it can be speculated that despite any preference for using coping strategies, depression cannot be prevented when the intensity of job stress is high.

## Conclusion

This study is an important step toward understanding the relationships between job stress and depression in Malaysian anti-drug professionals by taking into account individuals' various degrees of coping strategies. In this study, it was found that job stress was a crucial predictor of depression, regardless of what coping strategies were used. Still, control coping has good potential as a protective factor against depression in anti-drug professionals. While this study might carry some limitations (i.e., no causal-effect explained, used a self-report questionnaire only, and involved a single agency), its findings highlight the need to provide clinical supervision in correctional settings, which in this case, to assist anti-drug professionals in improving their attitudes, behaviors, and practices and achieve mental health well-being (51). Nevertheless, it is noteworthy to draw attention to these important aspects as a focus for the Malaysian government to formulate preventive initiatives for mental health issues in law enforcement agencies.

## Data availability statement

The raw data supporting the conclusions of this article will be made available by the authors, without undue reservation.

## Ethics statement

The studies involving human participants were reviewed and approved by Ethics Committee of Universiti Putra Malaysia. The participants provided their written informed consent to participate in this study.

## Author contributions

ND and MA contributed to the conception and design of the study. ND, NN, MR, and MA collected the data, organized the database, performed the statistical analysis, and wrote sections of the manuscript. All authors contributed to the manuscript revision and read and approved the submitted version.

## Funding

National Antidrug Agency (NADA) Research Grant (6300264).

## Conflict of interest

The authors declare that the research was conducted in the absence of any commercial or financial relationships that could be construed as a potential conflict of interest.

## Publisher's note

All claims expressed in this article are solely those of the authors and do not necessarily represent those of their affiliated organizations, or those of the publisher, the editors and the reviewers. Any product that may be evaluated in this article, or claim that may be made by its manufacturer, is not guaranteed or endorsed by the publisher.
