# Supplementary material for: Job stress and depression among Malaysian anti-drug professionals: The moderating role of job-related coping strategies
Source: Front Psychiatry. 2022 Nov 7;13:1020947. doi: 10.3389/fpsyt.2022.1020947 (PMC9676435; doi:10.3389/fpsyt.2022.1020947)
Supplement: Supplementary file 1 [file Table_1.DOCX]

**Supplementary Table 1** Regression analysis showing age, stress and control coping as predictors of depression (n = 3356)

|  | Depression | | | | |
| --- | --- | --- | --- | --- | --- |
| Predictor | B | β | *SE B* | *P* | *R^2^* /Δ*R^2^* |
| Model 1 |  |  |  |  | .01/ .01* |
| Age | -.07 | -.10 | .01 | .000 |  |
| Model 2 |  |  |  |  | .43/ .42* |
| Age | -.01 | -.02 | .01 | .142 |  |
| Stress | 4.05 | .64 | .08 | .000 |  |
| Control | -.55 | -.06 | .12 | .000 |  |
| Model 3 |  |  |  |  | .43/ .00* |
| Age | .01 | -.02 | .01 | .182 |  |
| Stress | 5.90 | .94 | .49 | .000 |  |
| Control | .58 | .07 | .32 | .069 |  |
| Stress x Control | -.45 | -.32 | .12 | .000 |  |

**p* < .001

Note: β = standardized regression coefficients; *SE* = Standard Error; p = significant value; Δ *R^2^*  = change in *R^2^* value
