# Supplementary material for: Job stress and depression among Malaysian anti-drug professionals: The moderating role of job-related coping strategies
Source: Front Psychiatry. 2022 Nov 7;13:1020947. doi: 10.3389/fpsyt.2022.1020947 (PMC9676435; doi:10.3389/fpsyt.2022.1020947)
Supplement: Supplementary file 2 [file Table_2.DOCX]

**Supplementary Table 2** Regression analysis showing age, stress and avoidant coping as predictors of depression (n = 3356)

|  | Depression | | | | |
| --- | --- | --- | --- | --- | --- |
| Predictor | B | β | *SE B* | *P* | *R^2^* /Δ*R^2^* |
| Model 1 |  |  |  |  | .01/.01 |
| Age | -.07 | -.10 | .01 | .000 |  |
| Model 2 |  |  |  |  | .43/.42 |
| Age | -.01 | -.02 | .01 | .122 |  |
| Stress | 3.75 | .60 | .09 | .000 |  |
| Avoidant | .83 | .11 | .12 | .000 |  |
| Model 3 |  |  |  |  | .44/.00 |
| Age | -.01 | -.02 | .01 | .134 |  |
| Stress | 2.28 | .36 | .30 | .000 |  |
| Avoidant | .35 | .05 | .26 | .179 |  |
| Stress x Avoidant | .47 | .34 | .09 | .000 |  |

**p* < .001

Note: β = standardized regression coefficients; *SE* = Standard Error; p = significant value; Δ *R^2^* = change in *R^2^* value
